# Use of Tin Foil by Junior Practitioners

**Published:** 1877-10

**Authors:** 


### USE OF TIN FOIL BY JUNIOR PRACTITIONERS.

"Of our thirty-two millions of people, less than half a million pay income tax, representing at the outside two millions of the population sufficiently well off to pay the usual fees for having their teeth stopped with gold; in other words, we find that only one person in sixteen can afford to do so. Yét it is generally conceded that gold is the only substance on which we can fully rely as a permanent stopping, and then only when it is introduced by an experienced hand, in a thoroughly conscientious manner. It therefore becomes a matter of very grave importance to every man whose lot it is to be a Dentist, to be able to use gold efficiently in those cases where he is called upon to do so; but how to attain that efficiency is the difficulty, for it can only be attained with great practice; probably the wisest plan for a man determined to get on would be to use gold in every case suited to it, although the patient could not afford to pay the cost of the foil. But struggling practitioners have not the time or the gold to do so, and the result is, in England, recourse is had to amalgam, but in America to tin foil, and to this cause more than all other causes put together, must be assigned the superior aptitude shown by the general run of American Dentists in the use of gold. Doubtless it will be urged that the manipulation of gold foil and tin foil are not identical; true, but both educate the mind and hand to laborious painstaking, so that the man who attains proficiency with the one, will not be very far behind with the other.

"On the other hand, one who habitually uses amalgam in cavities suited to gold, must give up all hope of ever attaining proficiency with gold, or taking a good position in his profession.

"A good stopping for the million is a thing yet to be discovered, but until that discovery is made, junior practitioners should make the most of tin foil."

The above, from the *British Journal of Dental Science*, presents some thoughts worthy of attention and serious consideration, not only by practitioners in England, but in America as well.

The criterion by which the writer estimates the proportion of the people able to procure the best operations for the preservation of their teeth, is hardly correct in the United States, however it may be in England.

In this country, many procure the best treatment and operations possible for their teeth, who pay no income tax nor any other tax. A true estimate of the importance and value of the teeth does not always correspond to wealth, culture, or position; some without either of these are most scrupulous in regard to the care of their teeth, and will be content with nothing less than the best operations upon them. Still the rule given is proximately true, perhaps, everywhere.

It is an important question, what shall be done for the fifteen sixteenths or even three-fourths of the people who are not able to procure the best treatment and operations for the preservation of their teeth.

Some practitioners adopt the plan of making every operation, with very few exceptions, as thorough as it is possible for them to do, and when the patient is not able to pay the usual fee, divide the expense; and success usually attends those who pursue this course; any other method tends to defective treatment for the patient, and demoralization of the dentist. This principle is well recognized by Dr. Carter, when he says, "One who habitually uses amalgam in cavities suited to gold, must give up all hope of ever attaining proficiency with gold, or taking a good position in his profession."

The correctness of this statement has been verified in a multitude of instances in this country. In some cases, those who were once superior operators with gold and tin foil, upon taking up the extensive use of amalgam, have become very inefficient in the use of the former; it not only works injury in this direction, but it destroys interest in the profession. It would be very gratifying indeed, if tin foil was used instead of amalgam in America as generally as is implied in the remark, "In England recourse is had to amalgam, but in America to tin foil." We suspect Dr. C. has a slight overflow of kindness, or does not wholly comprehend the exact state of the profession in America. We will hail the day as a bright one for the dental profession, when amalgam is superceeded by tin foil.

